# Erythroid lineage-specific lentiviral RNAi vectors suitable for molecular functional studies and therapeutic applications

**DOI:** 10.1038/s41598-022-13783-0

**Published:** 2022-08-18

**Authors:** Abhirup Bagchi, Nivedhitha Devaraju, Karthik Chambayil, Vignesh Rajendiran, Vigneshwaran Venkatesan, Nilofer Sayed, Aswin Anand Pai, Aneesha Nath, Ernest David, Yukio Nakamura, Poonkuzhali Balasubramanian, Alok Srivastava, Saravanabhavan Thangavel, Kumarasamypet M. Mohankumar, Shaji R. Velayudhan

**Affiliations:** 1 Center for Stem Cell Research (A Unit of inStem, Bengaluru, India), Christian Medical College, Vellore, Tamil Nadu 632002 India; 2 Department of Biotechnology, Thiruvalluvar University, Vellore, Tamil Nadu 632115 India; 3 Manipal Academy of Higher Education, Manipal, Karnataka 576119 India; 4 Sree Chitra Tirunal Institute for Medical Sciences and Technology, Thiruvananthapuram, Kerala 695011 India; 5 Department of Haematology, Christian Medical College, Vellore, Tamil Nadu 632004 India; 6 Cell Engineering Division, RIKEN BioResource Research Center, Ibaraki, 3050074 Japan

**Keywords:** Genetic transduction, Genetic vectors

## Abstract

Numerous genes exert multifaceted roles in hematopoiesis. Therefore, we generated novel lineage-specific RNA interference (RNAi) lentiviral vectors, H23B-Ery-Lin-shRNA and H234B-Ery-Lin-shRNA, to probe the functions of these genes in erythroid cells without affecting other hematopoietic lineages. The lineage specificity of these vectors was confirmed by transducing multiple hematopoietic cells to express a fluorescent protein. Unlike the previously reported erythroid lineage RNAi vector, our vectors were designed for cloning the short hairpin RNAs (shRNAs) for any gene, and they also provide superior knockdown of the target gene expression with a single shRNA integration per cell. High-level lineage-specific downregulation of *BCL11A* and *ZBTB7A, *two well-characterized transcriptional repressors of *HBG* in adult erythroid cells, was achieved with substantial induction of fetal hemoglobin with a single-copy lentiviral vector integration. Transduction of primary healthy donor CD34^+^ cells with these vectors resulted in >80% reduction in the target protein levels and up to 40% elevation in the γ-chain levels in the differentiated erythroid cells. Xenotransplantation of the human CD34^+^ cells transduced with H23B-Ery-Lin-sh*BCL11A* LV in immunocompromised mice showed ~ 60% reduction in BCL11A protein expression with ~ 40% elevation of γ-chain levels in the erythroid cells derived from the transduced CD34^+^ cells. Overall, the novel erythroid lineage-specific lentiviral RNAi vectors described in this study provide a high-level knockdown of target gene expression in the erythroid cells, making them suitable for their use in gene therapy for hemoglobinopathies. Additionally, the design of these vectors also makes them ideal for high-throughput RNAi screening for studying normal and pathological erythropoiesis.

## Introduction

Erythropoiesis is a multistep process in which hematopoietic stem and progenitor cells (HSPCs) differentiate through functionally and morphologically distinct stages of erythroid maturation to produce red blood cells (RBCs). Erythroid differentiation is tightly regulated by numerous factors, including cytokines, transcription factors, microRNAs (miRNAs) and signaling pathway proteins^1,2^. Recent studies on genome-wide occupancy of transcriptional factors and histone modifications have provided a critical understanding of the gene regulatory dynamics during different stages of erythroid differentiation^3,4^.

High-throughput screenings using RNA interference (RNAi) and gene editing using clustered regularly interspaced short palindromic repeats/Cas9 (CRISPR/Cas9) provide an unbiased approach to identify the genes involved in critical cellular and molecular processes. These strategies have pinpointed significant genes for erythropoiesis and globin gene switching^5–10^. However, RNAi using lentiviral vectors (LVs) with constitutive promoters to express shRNAs and CRISPR/Cas9-based gene editing that disrupts the target genes impair the maintenance and activity of all the hematopoietic cells in which these genes are functionally important. For example, downregulation of the expression of transcription repressor protein B-cell lymphoma/leukemia 11A (*BCL11A*) results in increased fetal hemoglobin (HbF) in adult erythroid cells. However, its deficiency causes delayed cell cycle entry and an aging-like phenotype in hematopoietic stem cells (HSCs)^11^. Using an LV with an erythroid lineage-specific promoter to express an shRNA to downregulates *BCL11A*, robust expression of HbF was achieved in the erythroid cells of the patients with sickle cell disease (SCD) and resulted in reduced sickling and increased half-life of their RBCs, without impairing HSCs and other lineages^12^.

Efficient RNAi is achieved with miRNA scaffold embedded shRNAs by RNA polymerase II promoters, which facilitate the expression of shRNAs similar to endogenous miRNAs^13–21^. Notably, the miRNA 30 (miR30) based scaffold has been used extensively to express the embedded shRNAs^14,21^. Recent advances in the design of highly potent shRNAs^14^ and modification of the miR30-based scaffolds - “miRE”^15,21^ and “UltramiR”^14^ for the increased shRNA biogenesis result in high-level knockdown of the target gene expression with a single copy LV integration of shRNAs. Significantly, these tools also minimize the off-target activity of the shRNAs^14,21^.

This study reports the generation of erythroid lineage-specific shRNA (Ery-Lin-shRNA) LVs with β-globin transcriptional regulatory elements, modified miR30 scaffolds and the most potent shRNAs for the robust downregulation of target gene expression. Two transcription factors, BCL11A and lymphoma and leukemia-related factor (LRF) encoded by zinc finger and BTB domain-containing protein 7A gene (*ZBTB7A*), have been recently identified as developmental stage-specific repressors that bind to specific genomic regions of β-globin cluster including *HBG* promoter to repress γ-globin expression in adult erythroid cells. We evaluated our LVs for the erythroid lineage-specific downregulation of these transcriptional repressors and demonstrated robust induction of HbF expression levels in erythroid cells in vitro and in vivo, without affecting cellular processes in other lineages. Unlike the previous erythroid lineage shRNA vector to express sh*BCL11A* currently being used for gene therapy for SCD^12,22,23^, our vectors allow cloning of any shRNA and, therefore, are suitable for the generation of pooled shRNA libraries for high-throughput screening of functionally significant genes in erythropoiesis. These Ery-Lin-shRNA LVs without selection markers or reporter genes may be further evaluated for their application in gene therapy for β-hemoglobinopathies by targeting various transcription regressors of *HBG* in adult erythroid cells.

## Methods

### Construction of lentiviral shRNA expression plasmids

The pZIP-MND-ZsGreen-UltramiR shRNA plasmid was generated by cloning the MND promoter sequence obtained from the pTRip-MND-GFP plasmid (kind gift from Francoise Pflumio) to replace the CMV promoter sequence at the *Cla*I and *Age*I restriction enzyme sites of pZIP-hCMV LV (Transomics Technologies Inc., Huntsville, AL, USA).

MSS-v1 and MSS-v5 lentiviral plasmids containing hypersensitivity sites (HSs) (kind gifts received from H. Trent Spencer) were used as the backbones for constructing H23B-Ery-Lin-shRNA and H234B-Ery-Lin-shRNA lentiviral plasmids. MSS-v1 and MSS-v5 contained HS2-HS3-HS4 and HS2-HS3 sequences, respectively. After digesting these plasmids with *Avr*II and *Cla*I, the PCR-amplified products of the β-globin promoter sequence from MSS-v5, the gene sequence for *Zoanthus* sp. green fluorescence protein (ZsGreen) from pZIP-MND and the mirE scaffold from the SGEP plasmid (Addgene #111170) were cloned by Gibson assembly using the NEBuilder HiFi DNA Assembly Cloning Kit (New England BioLabs, Ipswich, MA, USA). The generated vectors were named H23B-Ery-Lin-shRNA and H234B-Ery-Lin-shRNA, which contained HS2-HS3 and HS2-HS3-HS4 sequences, respectively.

For the construction of the H23BW-Ery-Lin-shRNA lentiviral plasmid, PCR amplified products of the β–globin promoter sequences from the MSS-v5 plasmid, the mirE scaffold from the SGEP plasmid, ZsGreen and the woodchuck post-transcriptional regulatory element (WPRE) from the pZIP-hCMV plasmid were cloned by Gibson assembly in the *Avr*II-*Cla*I digested MSS-v5.

### Selection and cloning of shRNAs

The sequences of the top-ranked shRNAs targeting *BCL11A* and *ZBTB7A* were obtained from the shERWOOD database (http://sherwood.cshl.edu:8080/sherwood/). The shRNA oligos were PCR-amplified and cloned into *Hpa*I-digested pZIP-MND-ZsGreen-UltramiR plasmid by Gibson assembly as reported earlier^14^ to generate pZIP–MND-UltramiR-sh*BCL11A*-1, -2 and -3 and pZIP-MND-UltramiR-sh*ZBTB7A*-1, -2 and -3 plasmids. The shRNA oligos were amplified with primers containing *Xho*I and *Eco*RI sites (Supplemental Table S1). The PCR products were digested with *Xho*I and *Eco*RI and cloned into H23B-Ery-Lin-shRNA, H234B-Ery-Lin-shRNA and H23BW-Ery-Lin-shRNA plasmids digested with the same enzymes.

### Cell culture

The HEK 293T, NB-4, THP-1 and HL-60 cell lines were purchased from American Type Culture Collection (ATCC, Manassas, Virginia, USA) and were cultured in Roswell Park Memorial Institute Medium 1640 (RPMI) (HyClone Laboratories, Logan, UT, USA) supplemented with 10% (vol/vol) fetal bovine serum (FBS) (HyClone Laboratories, Logan, UT, USA), 2 mM glutamine, 100 U/ml penicillin and 100 μg/ml streptomycin (all from Thermo Fisher Scientific, Grand Island, NY, USA).  HEK 293T cells were cultured in Dulbecco’s modified Eagle’s Medium (DMEM) (HyClone Laboratories, Logan, UT, USA) supplemented with 10% (vol/vol) fetal bovine serum (FBS), 2 mM glutamine, 100 U/ml penicillin and 100 μg/ml streptomycin(all from Thermo Fisher Scientific, Grand Island, NY, USA). The cultured cells were maintained at 37 °C in a humidified chamber supplemented with 5% CO_2_.

### Production of lentiviruses and titration

For the preparation of lentiviruses, the lentiviral shRNA expression plasmids were co-transfected into HEK 293T cells with pMD2.G envelope plasmid (Addgene #12259) and psPAX2 packaging plasmid (Addgene #12260) (gifts from Didier Trono) using FuGENE^®^ HD Transfection Reagent (Promega Corporation, Madison, WI, USA) following the manufacturer's protocols. The viral supernatants were collected after 48, 60 and 72 h, pooled, and concentrated by ultracentrifugation at 20,000 rpm for 2 h at 4 °C in a 70 Ti fixed angle rotor (Beckman Coulter, Palo Alto, CA). Aliquots of the viruses were frozen at − 80 °C until use.

### HUDEP-2 culture and differentiation

HUDEP-2 cells^24^ were cultured in StemSpan SFEM-II (StemCell Technologies, Vancouver, BC, Canada) supplemented with 1 μM dexamethasone (Sigma-Aldrich, St. Louis, MO, USA), 1 μg/ml doxycycline (Sigma-Aldrich), 2 mM L-glutamine, 100 U/ml penicillin and 100 μg/ml streptomycin (all from Thermo Fisher Scientific, Grand Island, NY, USA)  along with cytokines and growth factors as described earlier^24,25^. HUDEP-2 cells were differentiated as described earlier^26,27^.

### Flow cytometry

Flow cytometry for surface marker analysis was performed in the cells from ex vivo erythropoiesis with anti-CD71-APC (dilution 1:50) and anti-CD235a-PE (dilution 1:500) antibodies (BD Pharmingen, San Jose, CA, USA) as outlined previously^27^. The engraftment of human CD45^+^ cells was analysed using anti-hCD45 FITC (dilution 1:50) and anti-mCD45 APC (dilution 1:50) (BD Pharmingen). The multilineage potential of the engrafted cells was determined by staining 50,000 total BM cells with the suitable antibodies (Supplemental Table S2) (dilution 1:50 for all the antibodies). For HbF expression analysis, 50,000 terminally differentiated erythroid cells were centrifuged and washed with 200 μl of PBS with 0.1% BSA solution (PBS-B). The cells were fixed with 0.05% glutaraldehyde (MP Biomedicals, Solon, OH, USA) at the room temperature for 10 min followed by permeabilization with freshly prepared 0.1% Triton X-100 (Sigma-Aldrich) for 8 minutes. The cells were washed with PBS-B twice and incubated with anti-HbF APC antibody (Thermo Fisher Scientific, Camarillo, CA, USA) (dilution 1:25) in 50 μl of PBS-B at room temperature for 15–20 min. After washing the cells with PBS-B to remove the unbound antibodies, flow cytometry data acquisition was performed using CytoFLEX LX flow cytometer (Beckman Coulter, Indianapolis, IN, USA), and the results were analyzed using FlowJo 10.8.1 (FlowJO, Ashland, OR, USA). For the isolation of ZsGreen^+^ cultured erythroid cells, approximately 2X10^6^ total erythroid cells differentiated from the transduced HPSCs were washed with PBS and resuspended in 2 ml of Iscove’s Modified Dulbecco’s Medium (IMDM) (Thermo Scientific, Rockford, IL, USA) with 5% FBS, and the flow-sorting was performed using ARIA III flow cytometer (BD Biosciences, San Jose, CA, USA).

### HSPC isolation and expansion

Human granulocyte-colony stimulating factor (G-CSF)-mobilized CD34^+^ cells were isolated from the residual peripheral blood mononuclear cells (PBMNCs) of hematopoietic stem cell donors. The CD34^+^ HSPCs were isolated from the PBMNCs using EasySep™ Human CD34 positive selection kit (StemCell Technologies). HSPCs were expanded for 5 days using the HSPC expansion medium (HEM), which consisted of StemSpan SFEM-II containing 100 ng/ml stem cell factor (SCF), 100 ng/ml FMS-related tyrosine kinase 3 ligand (Flt3-L), 100 ng/ml thrombopoietin (TPO), 20 ng/ml interleukin-6 (IL-6), and 50 ng/ml interleukin-3 (IL-3) (all the cytokines purchased from Peprotech, Rocky Hill, NJ, USA), 2 mM glutamine, 100 U/ml penicillin and 100 μg/ml streptomycin (all from Thermo Fisher Scientific, Grand Island, NY, USA).

### Lentiviral transduction of human CD34^+^ HSPCs

Purified CD34^+^ HSPCs were seeded in HEM supplemented with 2 µM cyclosporin H (Sigma-Aldrich). After 24 h, CD34^+^ cells were transduced with Ery-Lin-shRNA LVs at a multiplicity of infection (MOI) of ~ 10 by spinfection and incubated for 24 h. The medium then was changed, and the cells were used for ex vivo erythropoiesis or mouse transplantation experiments.

### Ex vivo erythropoiesis

CD34^+^ HSPCs were expanded for 5 days in HEM and were subsequently differentiated to the erythroid lineage using the erythroid progenitor medium (EPM) containing StemSpan SFEM-II medium supplemented with 50 ng/ml SCF, 3 U/ml erythropoietin (Epo), 20 ng/ml IL-3, and 40 ng/ml insulin growth factor-1 (IGF-1) (all the cytokines and growth factors are purchased from Peprotech, Rocky Hill, NJ, USA), for 10–12 days. A previously described protocol was used with minor modifications to induce terminal erythroid differentiation^28^. Briefly, cultured erythroid cells were seeded at a density of 5 × 10^5^ cells/ml in erythroid differentiation medium-I (CD34-EDM-I) consisting of IMDM with Glutamax (ThermoFisher Scientific), 5% human AB serum (Sigma-Aldrich), 2 IU/ml heparin (Sigma-Aldrich), 10 μg/ml insulin (Sigma-Aldrich), 5 U/ml Epo (Peprotech), 500 μg/ml holotransferrin (Sigma-Aldrich) and 1 μM mifepristone (Sigma-Aldrich). On day 4, the cells were seeded at a density of 1 × 10^6^ cells/ml in CD34-EDM-II, which consisted of CD34-EDM-I without SCF, and were cultured with the medium change every other day until the end of differentiation. Total bone marrow (BM) cells isolated from NSG and NBSGW mice were also cultured using this four-step protocol described above for HSPC expansion and erythroid differentiation.

### Xenotransplantation of transduced CD34^+^ HSPCs into immunocompromised mice

NOD/LtSz-scid *Il2rg*^–/–^(NSG) mice (Jackson Laboratory, Bar Harbor, ME, USA) were irradiated with 2.5 Gy of γ-radiation 4 h before transplantation and NOD,B6.scid *Il2rg*^–/–^ *Kit*^*W41/W41*^ (NBSGW) mice (Jackson Laboratory) were preconditioned with busulphan (12.5 mg/kg body weight) for 48 h before transplantation. The H23BW-Ery-Lin-shRNA transduced and the untransduced CD34^+^ cells were administered at a dose of 1 × 10^6^ per mouse by tail-vein injection of female mice aged 8–12 weeks. Four mice were allotted for each experimental group; sh*BCL11A*, shScr and untransduced. Bone marrow (BM) cells were collected from the femur and tibia 16 weeks after the transplantation. Transplantation chimerism was evaluated after 8 weeks (short-term) and after 15–16 weeks (long-term) by flow cytometry analysis of human CD45 and mouse CD45 expression. The multilineage potential of the engrafted edited cells was determined by staining the cells with suitable antibodies (Supplemental Table S2).

The engraftment of human cells was calculated using the following equation

$$Engraftment\%=\frac{human\, {CD45}^{+}\,cells}{human\, {CD45}^{+}\,cells+mouse\, {CD45}^{+}\, cells}\times 100.$$

The animal experiments were conducted according to the ARRIVE guidelines^29^.

### High-performance liquid chromatography (HPLC) for globin chain analysis

Globin chain analysis using terminally differentiated erythroid cells was performed using a previously reported method^27^. The percentage of γ-globin chains was calculated as $\left(A\gamma +G\gamma \right)\%=\frac{A\gamma +G\gamma }{A\gamma +G\gamma +\beta }\times 100.$

### Quantitative real-time PCR analysis

Total RNA was extracted from cultured erythroid cells using RNAiso Plus (Takara Bio Inc., Kusatsu, Shiga, Japan), and 500 ng of total RNA was used for reverse transcription using the Primescript RT Reagent Kit (Takara Bio Inc.). Quantitative real-time PCR was performed with SYBR Premix Ex Taq II (Takara Bio Inc.) using specific primers (Supplemental Table S1) to assess the expression of *BCL11A* and *ZBTB7A*. Data were analyzed with QuantStudio 6 Flex real-time PCR systems (Applied Biosystems, Carlsbad, CA, USA).

### Western blot analysis

Western blot analysis was performed as described earlier^27^. Briefly, whole-cell lysates were prepared using radioimmunoprecipitation assay buffer containing phenylmethanesulfonylfluoride (Sigma-Aldrich) and Halt Protease Inhibitor Cocktail (Thermo Scientific, Rockford, IL, USA). The lysate (30 μg) was loaded on a 7% sodium dodecyl sulfate-polyacrylamide gel and the western blotting was performed using the primary antibodies, anti-BCL11A (1:1000 dilution) (Cell Signaling Technologies, Danvers, MA, USA), anti-LRF (1:1000 dilution) (Cell Signaling Technologies) and anti-actin (1:5000 dilution) (BD Pharmingen), and the secondary antibodies, anti-mouse IgG HRP (Cell Signaling Technologies) and anti-rabbit IgG HRP (Invitrogen Corporation, Camarillo, CA, USA).

### Vector copy number (VCN) analysis by ddPCR

Genomic DNA was extracted from cultured erythroid cells using the Gentra Puregene Cell Kit (Qiagen Sciences, Germantown, MD, USA) following the manufacturer’s protocol. The isolated DNA (1 μg) was digested by 40 units of *Hae*III (New England Biolabs) at 37 °C for 1 h. VCN was analyzed by droplet digital PCR (ddPCR)^30^ with ddPCR Supermix for Probes (Bio-Rad Laboratories, Hercules, CA, USA) with primers to amplify and quantitate the human immunodeficiency virus (HIV) sequence of the vector as the target region and human telomerase reverse transcriptase (hTERT) sequence as the endogenous control. The primer and probe sequences used for ddPCR are listed in Supplemental Table S1. The 22 µlPCR reaction mix was prepared for an automated Droplet Generator (Bio-Rad Laboratories) with final concentrations of primers and probe at 0.9 and 0.25 µM, respectively. After the droplet generation, PCR was performed using the following program: one cycle of 95 °C for 10 min; 40 cycles of 94 °C for 30 secs and 57 °C for 1 min; one cycle of 98 °C for 10 min; 4 °C hold. The PCR products were analyzed with a QX200 Droplet Reader (Bio-Rad Laboratories), and the results were quantitated using QuantaSoft (Bio-Rad Laboratories). VCN was calculated as 2× HIV copy/hTERT copy.

### Statistics

Data were analyzed by a 2-tailed t-test (95% confidence interval) using the GraphPad Prism software. *p ≤ 0.05, **p ≤ 0.01, ***p ≤ 0.001 ****p≤ 0.0001 were considered significant.

### Biosafety and ethics

All methods used in this study were carried out in accordance with the relevant guidelines and regulations. Lentiviral production was performed with the biosafety protocols approved by the institutional biosafety committee (IBSC) of Christian Medical College, Vellore. Experiments using PBMNCs and CD34^+^ HSPCs were approved by the Institutional Review Board (IRB) (Approval no: 10548) of Christian Medical College, Vellore. Written informed consents were obtained from the study subjects. The mice experiments were conducted following the guidelines and after approval by the institutional animal ethics committee (IAEC) of Christian Medical College, Vellore, India.

## Results

### Optimization of robust RNAi in erythroid cells

**Figure 1:**
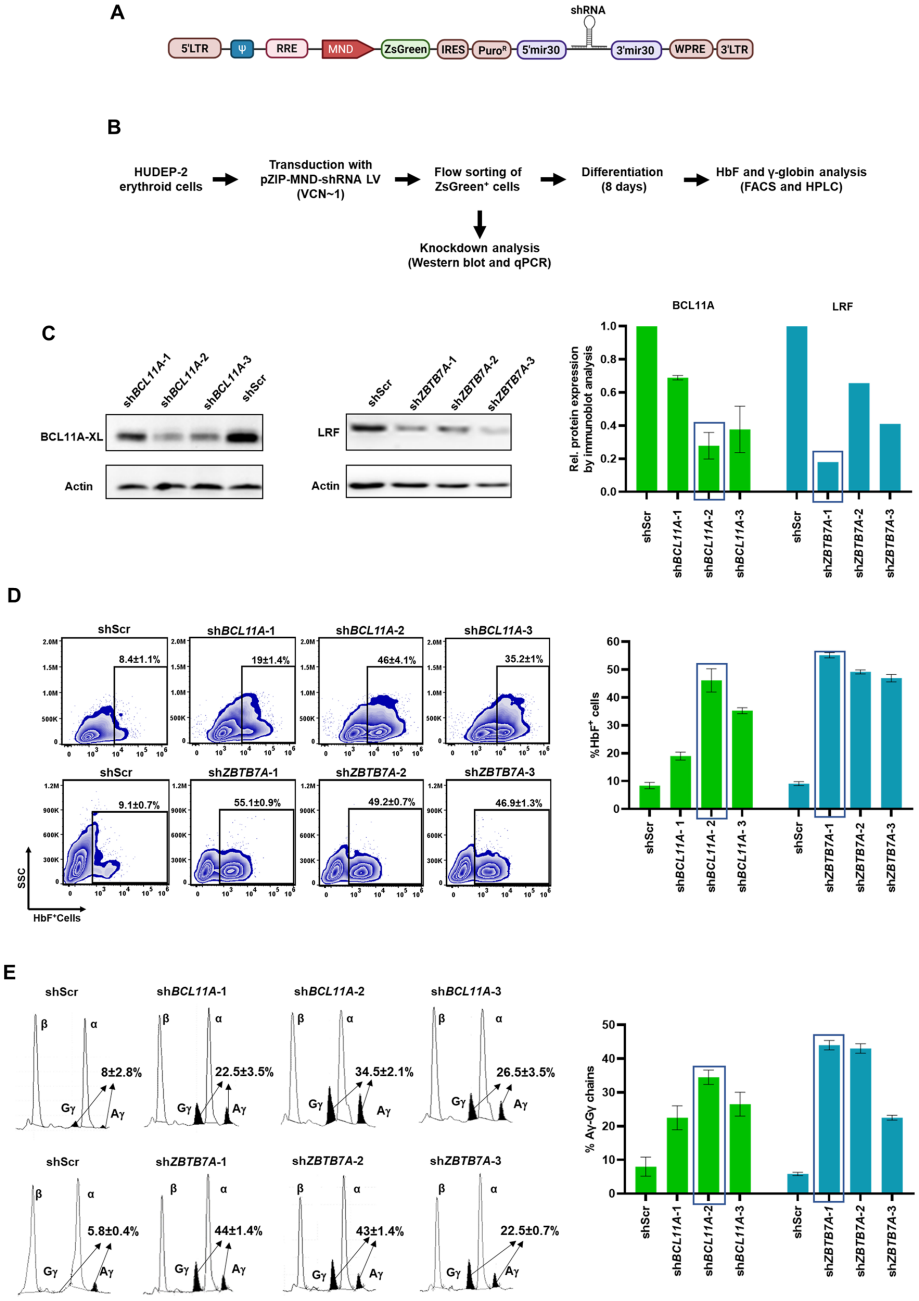
Ubiquitous knockdown of target gene expression in HUDEP-2 cells using pZIP-MND-ZsGreen-UltramiR shRNA LVs. (**A**) Schematic map of pZIP-MND-ZsGreen-UltramiR shRNA LV that co-expresses *Zoanthus* sp. green fluorescence protein (ZsGreen), puromycin resistance (PuroR) and an shRNA from the UltramiR scaffold under the MND promoter. 5′ miR 30 and 3′ miR30: 5′ and 3′ regions of the UltramiR shRNA scaffold, respectively. (**B**) Workflow of the experiment for the target gene knockdown experiment in the HUDEP-2 cells. (**C**) Representative immunoblot analysis to evaluate the knockdown efficiencies of the three shRNAs (labeled 1 to 3) that target BCL11A and compared to the scrambled shRNA (shScr) (data normalized to actin) (left) and the graphical representation of the densitometric data of the immunoblots (right). The highest knockdown efficiencies by sh*BCL11A* and sh*ZBTB7A* are indicated in boxes. Full blots are presented in Supplemental Fig. S9 and S10. (**D**) Intracellular HbF analysis by flow cytometry to determine the percentages of HbF^+^ cells in the shRNA transduced HUDEP-2 cells after differentiation (left) and graphical representation of the data (right). Boxes indicate the shRNAs with the highest percentages of HbF^+^ cells. (**E**) HPLC analysis of the percentages of Gγ+Aγ chains in the shRNA transduced cells after differentiation (left) and the graphical representation of the data (right). The shRNAs with the highest elevation in the percentages of Gγ+Aγ are indicated in boxes. All data represent mean ± SD (n = 2).

As knockdown of *BCL11A* or *ZBTB7A* causes an increase in HbF expression in erythroid cells that can be measured quantitatively by well-established methods^31–33^, we selected these two genes for the experiments to optimize an efficient RNAi strategy in erythroid cells. Three most potent shRNA sequences that target *BCL11A* (sh*BCL11A*-1, -2 and -3) and *ZBTB7A* (sh*ZBTB7A*-1, -2 and -3) (Supplemental Fig. S1) were designed using the shERWOOD algorithm^14^. The shRNAs were cloned into the “UltramiR” scaffold^14^ in the pZIP-MND-ZsGreen-UltramiR LV with MND (myeloproliferative sarcoma virus enhancer, negative control region deleted, dl587rev primer binding site substituted) promoter that confers stable and robust expression of transgenes or shRNAs in HSPCs and various hematopoietic lineages^34,35^. ZsGreen fluorescence protein, which is co-expressed with the shRNA from this vector facilitates the selection of the transduced cells for the subsequent experiments (Fig. 1A).

HUDEP-2 immortalized human erythroid progenitor cells^24^ were transduced separately with the LVs (Fig. 1B) to obtain 20-30% ZsGreen^+^ cells, with a single copy lentiviral integration per cell. sh*BCL11A*-2 and sh*ZBTB7A-*1 showed the highest knockdown of the target protein expression (> 70%) in the ZsGreen^+^ flow-sorted cells (Fig. 1C). For the functional characterization of the knockdowns, we measured the increase in the percentage of HbF^+^ cells and γ-globin chain expression in the transduced HUDEP-2 cells after terminal differentiation. Correlating with the knockdown efficiencies, sh*BCL11A*-2 showed the highest HbF expression with a 35.6–39.9% increase in the percentage of HbF^+^ cells (46 ± 4.1% in sh*BCL11A-*2 cells and 8.4 ± 1.1% in the cells with a scrambled control sequence [shScr]) (Fig. 1D and Supplemental Fig. S2A). Knockdown with sh*BCL11A*-2 also induced a 26–27% increase in γ-globin chain levels (34.5 ± 2.1% in sh*BCL11A-*2 cells and 8 ± 2.8% in shScr cells) (Fig. 1E and Supplemental Fig. S2B). Similarly, the knockdown levels of *ZBTB7A* by the three shRNAs inversely correlated with γ-globin levels. The sh*ZBTB7A*-1 showed a 44.9–47.2% (55.1 ± 0.65% in sh*ZBTB7A*-1 cells  and 9.1 ± 0.14% in shScr cells) increase in the percentage of HbF^+^ cells (Fig. 1D and Supplemental Fig. S2A) and a 37.5–38.8% (44 ± 1.4% in sh*ZBTB7A*-1 cells and 5.85 ± 0.49% in shScr cells) elevation in the γ-globin chain levels (Fig. 1E and Supplemental Fig. 2B). Our results validated that the highly potent shRNAs incorporated in the modified miR-shRNA scaffold of an LV with a robust constitutive promoter, such as the MND promoter, provide high-efficiency single-copy knockdown of the target gene expression in erythroid cells.

### LV construction for erythroid lineage-specific knockdown

**Figure 2:**
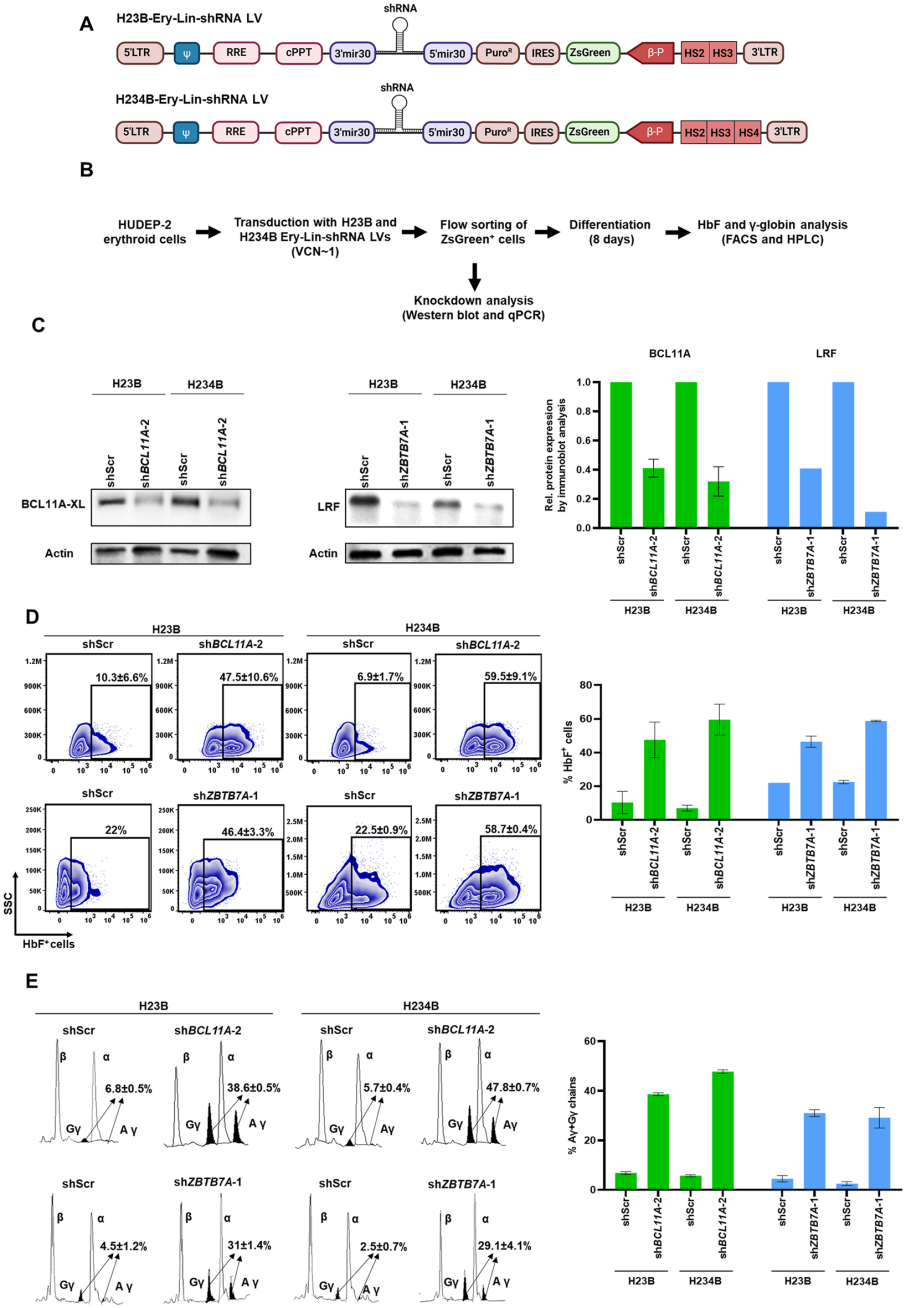
Knockdown of target gene expression in HUDEP-2 cells using H23B and H234B Ery-Lin-shRNA LVs. (**A**) Schematic maps of H23B and H234B Ery-Lin-shRNA LVs. HS2, HS3 and HS4: human β-globin LCR hypersensitivity sites 2, 3 and 4, respectively, βp: β-globin promoter and 5' miR30 and 3' miR30: 5′ and 3′ regions of the mir30 scaffold sequence, respectively. (**B**) Workflow of the experiment for target gene knockdown by the Ery-Lin-LVs in HUDEP-2 cells. (**C**) Representative immunoblots to evaluate the knockdown efficiencies of sh*BCL11A* and sh*ZBTB7A* in the transduced HUDEP-2 cells (left) and the graphical representation of the densitometric data of the immunoblots (right). Full blots are presented in Supplemental Fig. S9 and S10. (**D**) Intracellular HbF analysis by flow cytometry to determine the percentages of HbF^+^ cells in the shRNA transduced HUDEP-2 cells after differentiation. (**E**) HPLC analysis of the percentages of Gγ+Aγ chains in the shRNA transduced cells after differentiation (left) and graphical representation of the data (right). H23B and H234B represent H23B-Ery-Lin-shRNA and H234B-Ery-Lin-shRNA LVs, respectively. All data represent mean ± SD (n = 2).

Downregulation of target gene expression using shRNAs expressed from constitutive promoters, such as MND, is unsuitable for studying lineage-specific functions. Instead, the erythroid lineage-specific knockdown of the target gene expression can be achieved successfully using an LV that contains the miR-shRNA cassette under the transcriptional control of a β-globin proximal promoter linked to the hypersensitive sites 2 and 3 (HS2 and HS3) of the β-globin locus control region (LCR)^22,23^. The β-globin promoter exhibits high-level erythroid-specific expression and has been widely used for expressing globin genes for SCD gene therapy^36,37^. We generated two erythroid lineage-specific shRNA (Ery-Lin-shRNA) LVs, H23B-Ery-Lin-shRNA and H234B-Ery-Lin-shRNA (Fig. 2A), which contain “miRE”-scaffold^21^ with increased shRNA biogenesis potential and restriction enzyme sites that facilitate the cloning of shRNAs. Similar to the erythroid-lineage shRNA vector reported previously^23^, our Ery-Lin-shRNA LVs contain the human β-globin promoter and LCR-HSs that enable the erythroid-specific expression of shRNA. H23B-Ery-Lin-shRNA LV contained HS2 (1097 bp) and HS3 (844 bp) elements and HS234B-Ery-Lin-shRNA LV contained HS4 (1243 bp) in addition to HS2 and HS3 elements.

The knockdown efficiencies of the Ery-Lin-shRNA LVs were evaluated by transducing HUDEP-2 cells with the LVs containing sh*BCL11A*-2 and sh*ZBTB7A*-1, the two shRNAs with the highest knockdown efficiencies, separately at a low transduction efficiency (20–30%) to maintain approximately single copy LV integration (Fig. 2B and Supplemental Fig. S3). ZsGreen fluorescence reporter protein co-expressed with the shRNA in these vectors facilitated the selection of transduced cells for the subsequent experiments. The ZsGreen^+^ flow-sorted cells that contained single shRNA integration events showed efficient knockdown of BCL11A and LRF expression (Fig. 2C). Furthermore, the percentage of HbF^+^ cells in the terminally differentiated transduced HUDEP-2 cells increased by 37.2 ± 3.9%, 52.5 ± 10.9%, 24.4 ± 3.3%, and 36.3 ± 0.7% with H23B-Ery-Lin-sh*BCL11A*-2, H234B-Ery-Lin-sh*BCL11A*-2, H23B-Ery-Lin-sh*ZBTB7A*-1 and H234B-Ery-Lin-sh*ZBTB7A*-1, respectively, compared to the corresponding shScr controls (Fig. 2D).  The mean γ-globin induction levels in the differentiated ZsGreen^+^cells were 38 to 48 % by the two Ery-Lin-sh*BCL11A*-2 LVs and ~30% by the Ery-Lin-sh*ZBTB7A*-1 LVs (Fig. 2E). Comparison of the ZsGreen expression by H234B-Ery-Lin-shScr and H23B-Ery-Lin-shScr LVs showed that H234 LV exhibited relatively higher promoter activity than H23B LV (Supplemental Fig. S4A), but the latter generated a 10-fold higher titer than the former.

We assessed the erythroid specificity of H23B-Ery-Lin-shScr LV by analyzing ZsGreen expression in non-erythroid cells. Three myeloid cell lines (NB4, THP-1 and HL60-1) and purified αβ T -cells along with HUDEP-2 cells were transduced with a mixture of lentiviruses generated by H23B-Ery-Lin-shRNA and pLKO5-sgRNA-EFS-tRFP LVs. We observed expression of red fluorescence protein from pLKO5-sgRNA-EFS-tRFP LV by the human elongation factor 1α short (EFS) promoter in all the four non-erythroid cell types and HUDEP-2 erythroid cells, but ZsGreen expression was detected only in HUDEP-2 cells (Supplemental Fig. S5). These results confirmed that the expression of H23B-Ery-Lin-shRNA-LV is transcriptionally inactive in non-erythroid lineages, such as myeloid and lymphoid cells; hence, this vector  is suitable for erythroid-specific knockdown of target gene expression.

### Evaluation of modified Ery-Lin-shRNA LV in erythroid cells differentiated from CD34^+^ HSPCs

**Figure 3:**
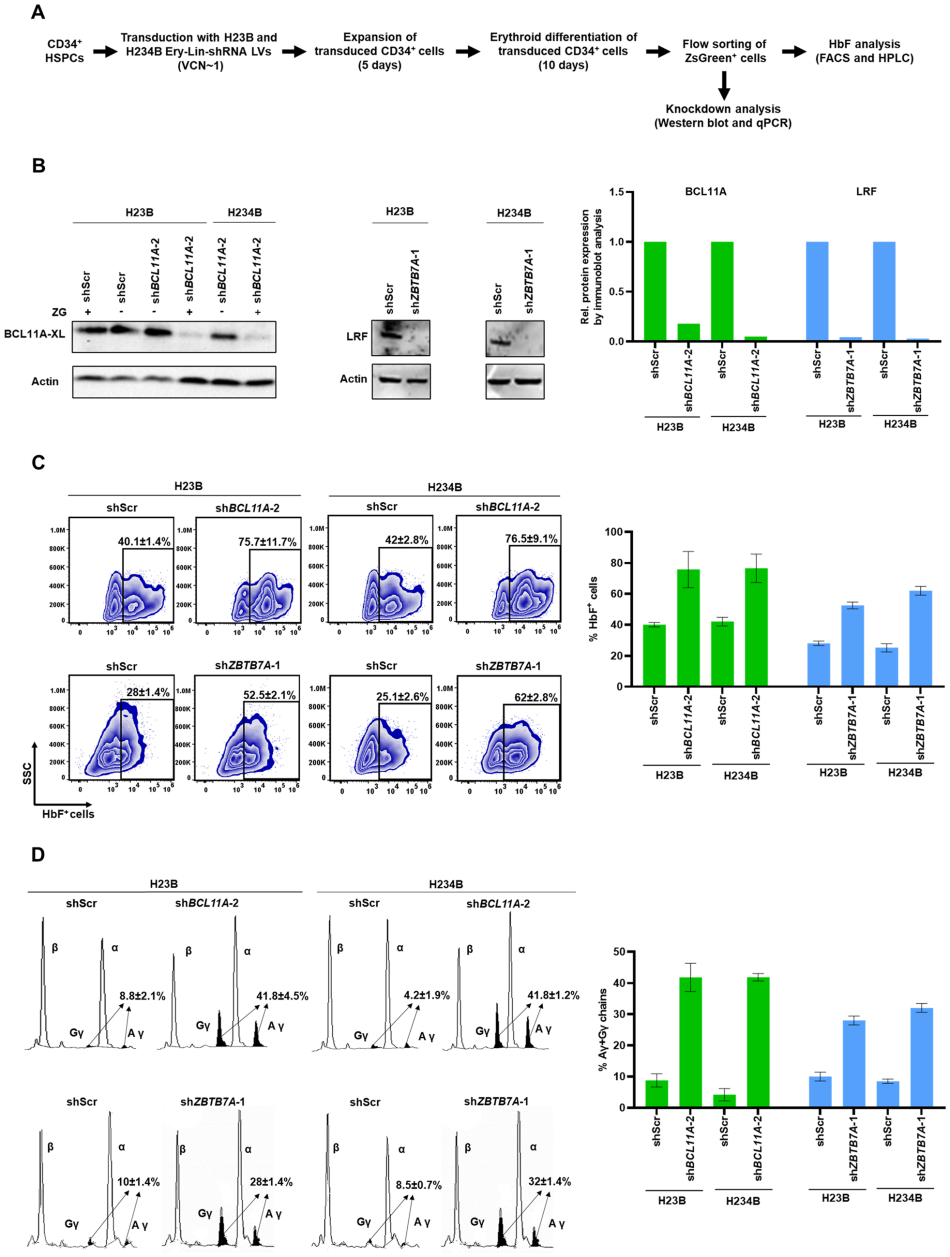
Erythroid-specific downregulation of target gene expression in the erythroid cells differentiated from CD34^+^ HSPCs transduced with Ery-Lin-shRNA LVs. (**A**) Schematic outline of the experiment. (**B**) Representative immunoblots that illustrate the knockdown efficiencies of sh*BCL11A* and sh*ZBTB7A* in the erythroid cells (left) and the densitometric data of the immunoblots (right). ZG represents ZsGreen expression; ‘+’ indicates ZsGreen^+^ cells and ‘–’ indicates ZsGreen^-^ cells. Full blots are presented in Supplemental Fig. S9 and S10. (**C**) Intracellular HbF analysis by flow cytometry to determine the percentages of HbF^+^ cells in the erythroid cells differentiated from the shRNA transduced human CD34^+^ HSPCs  (left) and the graphical representation of the data (right). (**D**) HPLC analysis of the percentages of Gγ+Aγ chains in the shRNA transduced cells after differentiation (left) and the graphical representation of the data (right). H23B and H234B represent H23B-Ery-Lin-shRNA and H234B-Ery-Lin-shRNA LVs, respectively. All data are mean ± SD (n = 2).

CD34^+^ HSPCs undergo progressive changes in the transcriptional, epigenetic and proteomic profiles during differentiation into the erythroid lineage. The different cellular stages during erythroid differentiation may be monitored using specific surface markers expressed in the early and late stages of differentiation. CD34^+^ HSPCs transduced with Ery-Lin-shRNA LVs may be suitable for studying the effect of knockdown of target gene expression on erythroid maturation. We transduced HSPCs with H23B-Ery-Lin-shRNA and H234B-Ery-Lin-shRNA LVs encoding sh*BCL11A*-2, sh*ZBTB7A*-1 and shScr (Fig. 3A). ZsGreen^+^CD71^+^ cells comprised 25–35% of the population on day 7 of ex vivo erythroid differentiation confirmed low copy number integration of the Ery-Lin-shRNA LVs. The ZsGreen^+^ erythroid cells were flow-sorted, expanded and further differentiated towards later stages of erythropoiesis**.** The steady increase in ZsGreen expression level as the the cells underwent gradual erythroid maturation forming a large number of CD71^+^CD235a^+^ cells suggested the constant activation from the transcription regulation unit of Ery-Lin-shRNA LVs during erythroid differentiation. We observed >80% knockdown of BCL11A and LRF in the cells on day 12 of erythroid differentiation (Fig. 3B). The knockdown of these gene expression resulted in 4 to 10 fold upregulation of *HBG* mRNA expression (Supplemental Fig. S6). Both H23B- and H234B- Ery-Lin-shRNA LVs caused a significant increase in the percentage of HbF^+^ cells and γ-globin chain levels compared to the respective shScr (Fig. 3C and D).

HbF and γ-globin expression levels in the erythroid cells derived from the transduced HSPCs suggest that our Ery-Lin-sh*BCL11A* LV is comparable to the current sh*BCL11A* LV is used for gene therapy^12,22^. Lineage-specific knockdown of *ZBTB7A* has not been tested previously for its ability to increase HbF in the primary human erythroid cells. In this study, we observed a ~ 30% increase in the percentages of HbF^+^ cells after erythroid-specific knockdown of *ZBTB7A*. As reported earlier^31^, we also observed a modest defect in terminal erythroid differentiation after *ZBTB7A* knockdown (Supplemental Fig. S7). Based on these results, we conclude that H23B- and H234B-Ery-Lin-shRNA LVs are suitable for downregulating any target gene to study its erythroid lineage functions due to the feasibility of cloning shRNAs at the restriction enzyme sites in the mirE scaffold in these LVs.

### H23B-Ery-Lin-shRNA gene-modified human CD34^+^ HSPCs exhibit erythroid lineage-specific knockdown in immunocompromised mice

**Figure 4:**
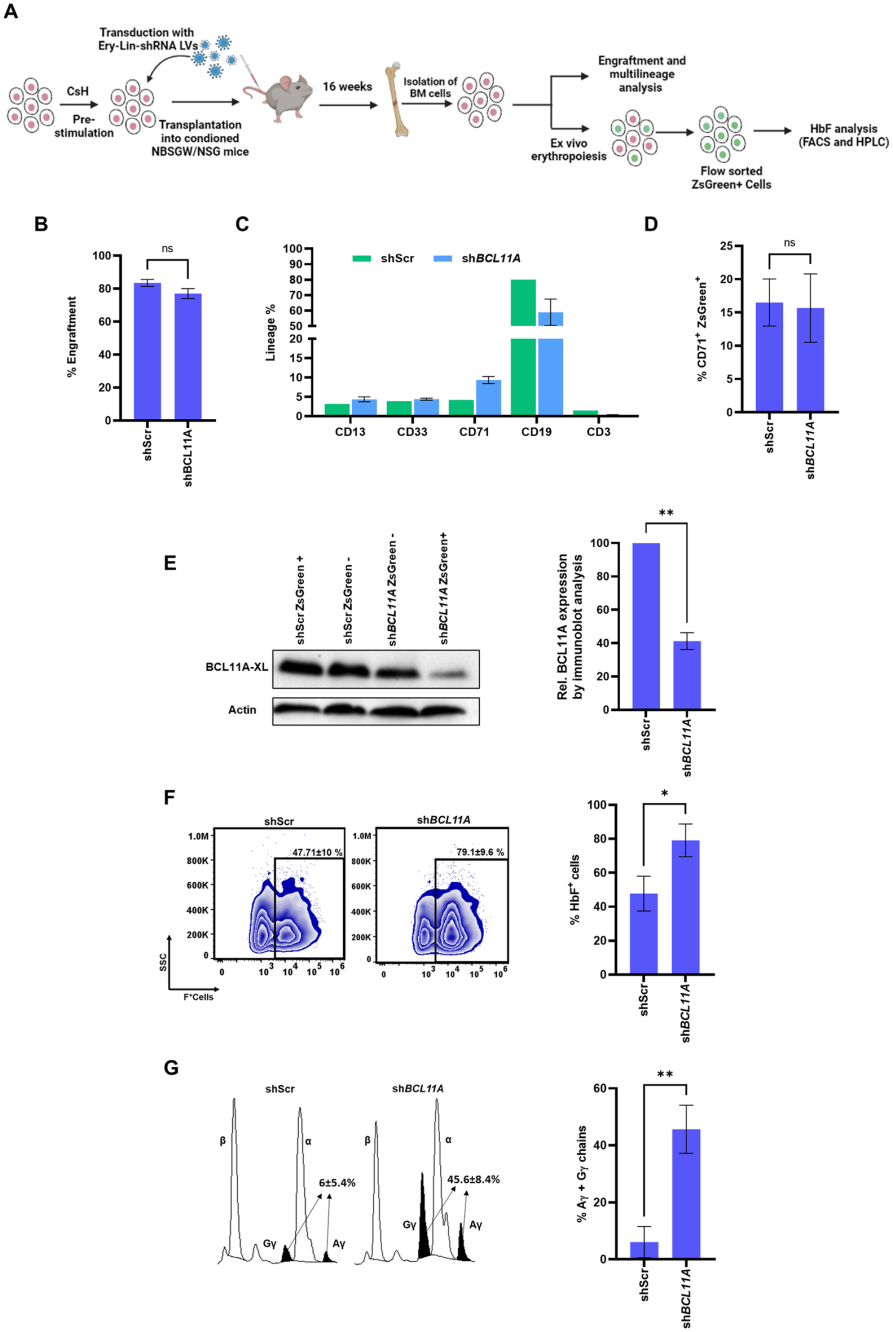
Analysis of the engrafted H23BW-Ery-Lin-sh*BCL11A* LV transduced HSPCs in NBSGW mice. (**A**) Schematic representation of the mouse transplantation experiment. CsH: cyclosporin H. (**B**) The percentage engraftment measured in the total BM cells isolated 15 weeks after transplantation. (**C**) Multilineage analysis in the human CD45 positive (hCD45^+^) cells after 15 weeks of transplantation. (**D**) The percentage of CD71^+^ ZsGreen^+^cells in the erythroid cells obtained from BM by ex vivo erythropoiesis. (**E**) Representative immunoblots (left) and the densitometric quantitation of BCL11A knockdown (right) in the flow-sorted ZsGreen^+^ erythroid cells (normalized to actin levels). (**F**) Intracellular HbF analysis by flow cytometry to determine the percentages of HbF^+^ cells in the terminally differentiated ZsGreen^+^ erythroid cells (left) and the graphical representation of the data (right). (**G**) HPLC analysis of the percentages of Gγ+Aγ chains in the terminally differentiated ZsGreen^+^ erythroid cells after differentiation (left) and the graphical representation of the data (right). All data are compared to shScr. All data are mean ± SD (n = 3). ns, not significant, *p < 0.05 and ** *p* < 0.01.

We performed xenotransplantation experiments to test the long-term engraftment potential of the Ery-Lin-shRNA LV transduced CD34^+^ HSPCs and the long-term erythroid lineage-specific knockdown of the target gene expression. In our experiments with the transduced CD34^+^ cells and ex vivo erythropoiesis, we observed that the knockdown efficiencies by H23B- and H234B-Ery-Lin-shRNA LVs were not significantly different (Fig. 3B), and  the inclusion of HS4 element in the LVs has been reported to reduce viral titers^36,37^.Therefore, we used H23B-Ery-Lin-shRNA LV for the mouse transplantation experiments. We incorporated the woodchuck post-transcriptional regulatory element (WPRE) into this vector as it enhances the transgene expression^38–40^. We carried out the mouse transplantation experiments with sh*BCL11A* LV to study its knowdown effect in vivo as *ZBTB7A* knockdown resulted in erythroid maturation defects in our ex vivo erythropoiesis experiments (Supplemental Fig. S7). The CD34^+^ HSPCs from a healthy donor were transduced with H23BW-Ery-Lin-sh*BCL11A-*2 and H23BW-Ery-Lin-shScr LVs at an MOI of ~ 10 and were transplanted into NOD/B6/SCID/IL-2rγ^−/−^ Kit^W41/W41^ (NBSGW) mice, which display enhanced engraftment of human HSPCs^41,42^ and also support erythropoiesis of transplanted human CD34^+^ cells more efficiently than NOD.Cg-Prkdc^scid^ Il2rg^tm1Wjl^/SzJ (NSG) mice^43^ (Fig. 4A). BM cells were harvested and evaluated for engraftment and multilineage analysis 15 weeks after transplantation. We observed similar engraftment levels in the sh*BCL11A* (98.56 ± 0.7%) and shScr (96.95 ± 2.47%) groups, suggesting that sh*BCL11A* from our Ery-Lin-shRNA LV does not affect the survival and engraftment of HSPCs (Fig. 4B). Multilineage repopulation analysis of CD13^+^ CD33^+^ myeloid cells, CD71^+^ erythroid cells, CD19^+^ B-cells and CD3^+^ T-cells in the human CD45^+^ population showed that *BCL11A* knockdown did not cause lineage bias in the reconstituted hematopoiesis (Fig. 4C). Selective expansion of erythroid progenitors from the total BM cells showed 10–20% (15.6 ± 5.1%) CD71^+^ ZsGreen^+ ^human erythroid cells after 5 days of culture (Fig. 4D). We observed a 54–64% (59 ± 5.03%) reduction in the BCL11A protein levels in the sh*BCL11A* group compared to the shScr group (Fig. 4E). The VCN using ddPCR in the sorted ZsGreen^+^ cells was 0.9–1 in both the sh*BCL11A* and shScr groups. The terminally differentiated ZsGreen^+^ cells were analyzed for the percentages of HbF^+^ cells and γ-globin chains. There was a 35–36% (33.8 ± 2%) increase in HbF^+^ cells (Fig. 4F). There was also a significant increase in γ-globin chain levels (~40%) in the sh*BCL11A* group compared to the shScr group (Fig. 4G). H23BW-Ery-Lin-shRNA LV in NSG mice showed comparable engraftment levels after 8 or 16 weeks of transplantation (Supplemental Fig. S8A). Cultured erythroid cells from STE-LTE- BM showed a significant elevation in the percentage of HbF^+^ erythroid cells derived from mice transplanted with the sh*BCL11A* transduced CD34^+^ cells  compared to shScr (Supplemental Fig. S8E). These mouse transplantation experiments revealed that H23BW-Ery-Lin-shRNA LV provides a robust erythroid-specific expression of shRNAs without affecting the long-term multilineage potential of the transduced human HSPCs. The ability of H23BW-Ery-Lin-shRNA LV to increase HbF in the human erythroid cells in the mice transplanted with transduced human CD34^+^ cells suggests the potential application of this LV in the therapeutic reactivation of HbF for β-hemoglobinopathies. Moreover, restriction enzyme sites that allow the cloning of oligonucleotide pools makes this vector suitable for high-throughput studies to identify the genes involved in human erythropoiesis by mouse transplantation experiments.

## Discussion

Post-transcriptional downregulation of genes by RNAi has been used effectively for probing the transcriptional regulatory mechanisms of erythropoiesis^3,8,10,44^. Widely used constitutive Pol II promoters, such as spleen focus forming virus and human cytomegalovirus promoters, undergo epigenetic silencing during cellular differentiation^45,46^, whereas MND promoter confers stable and robust expression in HSPCs and various hematopoietic lineages^34,35^. However, lineage or cell-specific gene knockdown provides unambiguous phenotypic effects in specific cell populations without affecting the target gene expression in other cell types. Previous studies have shown that this could be achieved using cell/lineage-specific promoters^23,47,48^. The lack of an erythroid lineage-specific LV that facilitates easy cloning of shRNAs has been a hurdle for high-throughput screening using RNAi libraries for the comprehensive screening of genes involved in erythropoiesis. In this study, we constructed universal LVs with a β-globin promoter combined with β-globin LCR sequences for lineage-specific expression of shRNAs that provide high knockdown efficiencies in erythroid cells.

We first optimized robust RNAi conditions in erythroid cells using an LV with a constitutive MND promoter and “UltramiR” miR-shRNA scaffold and the most potent shRNAs selected using the shERWOOD algorithm, which confers increased shRNA expression and target gene knockdown levels^14,21^. We chose *BCL11A* and *ZBTB7A* as our target genes to evaluate the knockdown efficiency as the decrease in expression levels of these transcription repressors in adult human erythroid cells results in elevated HbF levels, which can be easily quantitated experimentally. We observed that the modified miR-shRNA scaffold embedded with robust shRNAs provided significant downregulation of the target gene expression in erythroid cells.

The architecture of our Ery-Lin-LVs was framed with several modifications required for the robust expression of shRNAs for efficient knockdown of any gene that is transcriptionally active during erythropoiesis, with a single copy LV integration per cell. First, to regulate erythroid specificity, we positioned the shRNAs to be expressed under the control of the minimal core β-globin promoter and β-globin LCR HS2, HS3 and HS4 elements. Since the inclusion of HS4 drastically reduces viral titers^37,50^, without a significant difference in ZsGreen expression (Supplemental Fig. S4A), we carried out our mouse transplantation experiments with H23BW-Ery-Lin-shRNA LV. Our study concluded that the HS4 LCR element was dispensable for erythroid lineage-specific transgene expression. Second, the H23BW-Ery-Lin-shRNA LV generated by incorporating the WPRE sequence further enhanced transgene expression with consistent and robust shRNA activity both in vitro and in vivo. In addition to these adaptations, we designed our vector with restriction enzyme sites incorporated within the miR-shRNA scaffold simplifing the cloning of any shRNA.

Activation of HbF by downregulating the expression of transcription repressors of *HBG* is an effective strategy for the treatment of patients with SCD and TM^12,49,51^, the two most common monogenic human disorders that affect the expression or function of β-globin gene expressed in the adult stage erythroid cells. Knockdown of the γ-globin repressors, BCL11A and  LRF*,* results in a significant elevation of HbF level in adult human erythroid cells^22,33,52^. Ubiquitous downregulation of *BCL11A* delays cell cycle entry and an aging-like phenotype in HSCs^11^, and *ZBTB7A* leads to differentiation defects in myeloid and lymphoid lineages^53,54^. However, erythroid lineage-specific knockdown of *BCL11A* has been shown to circumvent the toxicity of its downregulation in other hematopoietic cell types^23^. We also obtained erythroid-specific downregulation of BCL11A and LRF using our Ery-Lin-LVs, which resulted in a significant increase in the expression of γ-globin and HbF in adult erythroid cells. Xenotransplantation of CD34^+^ cells transduced with sh*BCL11A* achieved up to 40% HbF with single-copy shRNA integration. As reported earlier^23,31,55^, we did not observe any adverse effect of *BCL11A* downregulation in erythroid maturation, while *ZBTB7A* downregulation resulted in erythroid differentiation defects.

In conclusion, the Ery-Lin-shRNA LVs that we report here exhibit robust erythroid-specific knockdown of target gene expression for their functional characterization in erythropoiesis. An important application of our LVs is RNAi screening in erythroid cells, as they allow the cloning of oligonucleotide pools to generate RNAi libraries. The robust long-term erythroid-specific expression without the significant silencing of the expression of shRNAs after xenotransplantation of transduced human CD34^+^ cells in NBSGW mice demonstrates the suitability of these LVs for performing in vivo RNAi screening experiments for studying erythropoiesis. A significant increase in HbF levels in the erythroid cells derived from the CD34^+^ cells transduced with Ery-Lin-sh*BCL11A* LVs suggests that this vector may be suitable for gene therapy applications for hemoglobinopathies after modifications to remove the selection markers.

## Supplementary Information

Supplementary Information.
