# Supplementary material for: Erythroid lineage-specific lentiviral RNAi vectors suitable for molecular functional studies and therapeutic applications
Source: Sci Rep. 2022 Aug 18;12:14033. doi: 10.1038/s41598-022-13783-0 (PMC9388678; doi:10.1038/s41598-022-13783-0)
Supplement: Supplementary file 1 — Supplementary Information. [file 41598_2022_13783_MOESM1_ESM.pdf]

Supplemental information

Erythroid lineage-specific lentiviral RNAi vectors suitable for molecular functional studies and therapeutic applications

Abhirup Bagchi[1,2 †], Nivedhitha Devaraju[1,3 †], Karthik Chambayil[1,4], Vignesh Rajendiran[1,4], Vigneshwaran Venkatesan[1,3], Nilofer Sayed[1], Aswin Anand Pai[4,5], Aneesha Nath[1,4], Ernest David[2], Yukio Nakamura[6], Poonkuzhali Balasubramanian[4,5], Alok Srivastava[1,4,5], Saravanabhavan Thangavel[1,3], Kumarasamypet M Mohankumar[1,3*], Shaji R Velayudhan[1,2,5*]

[1]Center for Stem Cell Research (A Unit of inStem, Bengaluru, India), Christian Medical College, Vellore, Tamil Nadu-632002, India.

[2]Department of Biotechnology, Thiruvalluvar University, Vellore, Tamil Nadu-632115, India.

[3]Manipal Academy of Higher Education, Manipal, Karnataka-576119, India.

[4]Sree Chitra Tirunal Institute for Medical Sciences and Technology, Thiruvananthapuram, Kerala 695011, India.

[5]Department of Haematology, Christian Medical College, Vellore, Tamil Nadu-632004, India.

[6]Cell Engineering Division, RIKEN BioResource Research Center, Ibaraki-305-0074, Japan.

[†]These authors equally contribute as first authors

*These authors jointly supervised this work.

Correspondence and requests for materials should be addressed to S.R.V (email: rvshaji@cmcvellore.ac.in) and K.M.M (mohankumarkm@cmcvellore.ac.in).

Supplemental Figures

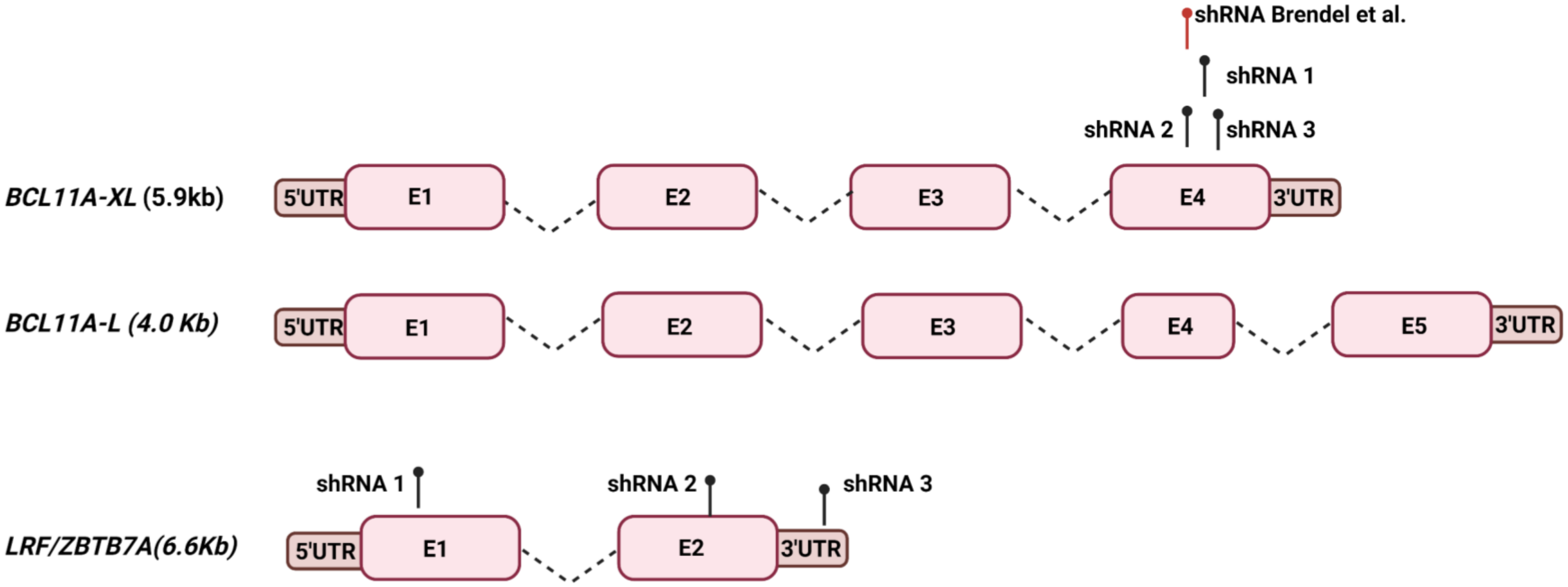

Supplemental Fig. S1. The positions of the selected shRNAs (1, 2 and 3) that target the two *BCL11A* isoforms (XL and L) and *ZBTB7A*. E1, E2, E3, E4 and E5 indicate exons 1, 2, 3, 4 and 5, respectively. The position of previously described sh*BCL11A* by Brendel et al. [1] is also shown.

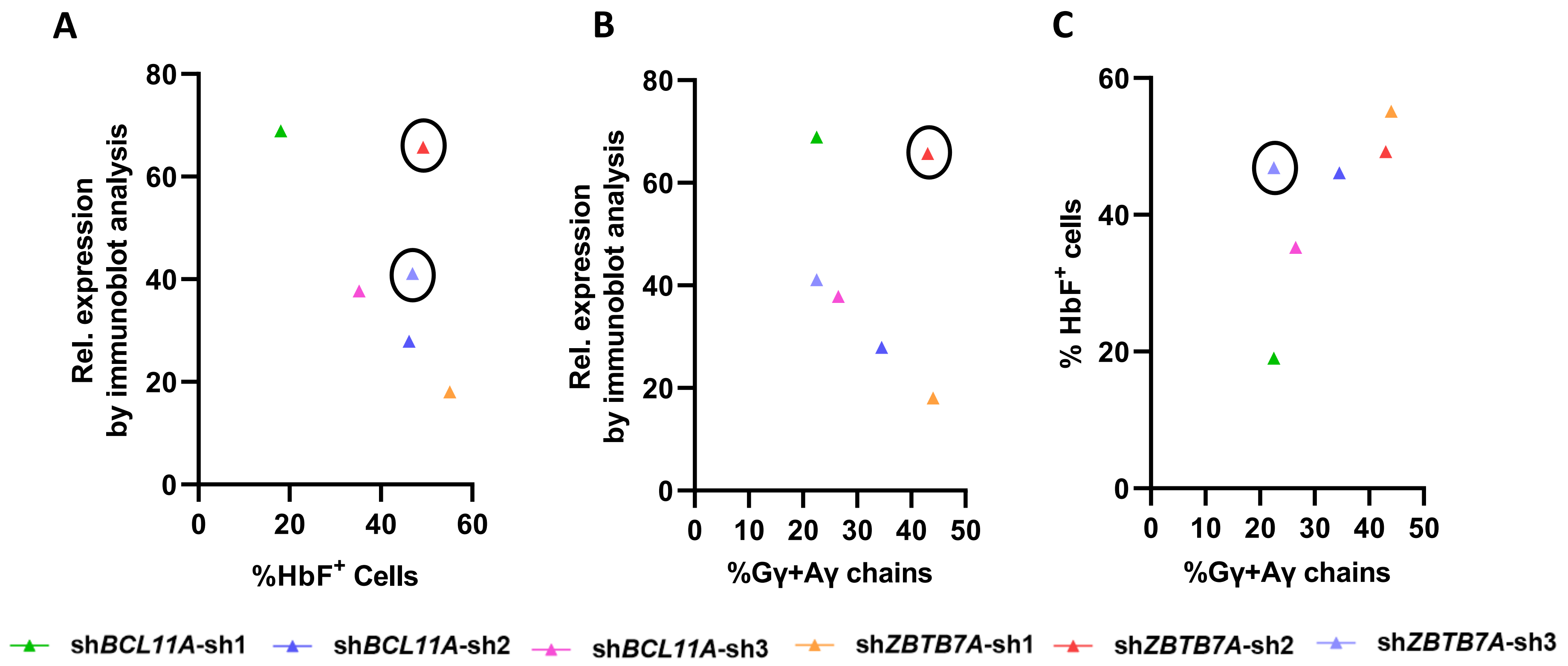

Supplemental Fig. S2. (A) Correlation between the knockdown efficiencies of the *BCL11A* and *ZBTB7A* shRNAs and the percentages of HbF^+ cells. (B) Correlation between the knockdown efficiencies of the shRNAs and the percentages of Gγ+Aγ chains. (C) Correlation between the percentages of HbF^+ cells and the percentages of Gγ+Aγ chains. Each value on the graph signifies the mean of two independent experiments. Black circles represent the outliers.

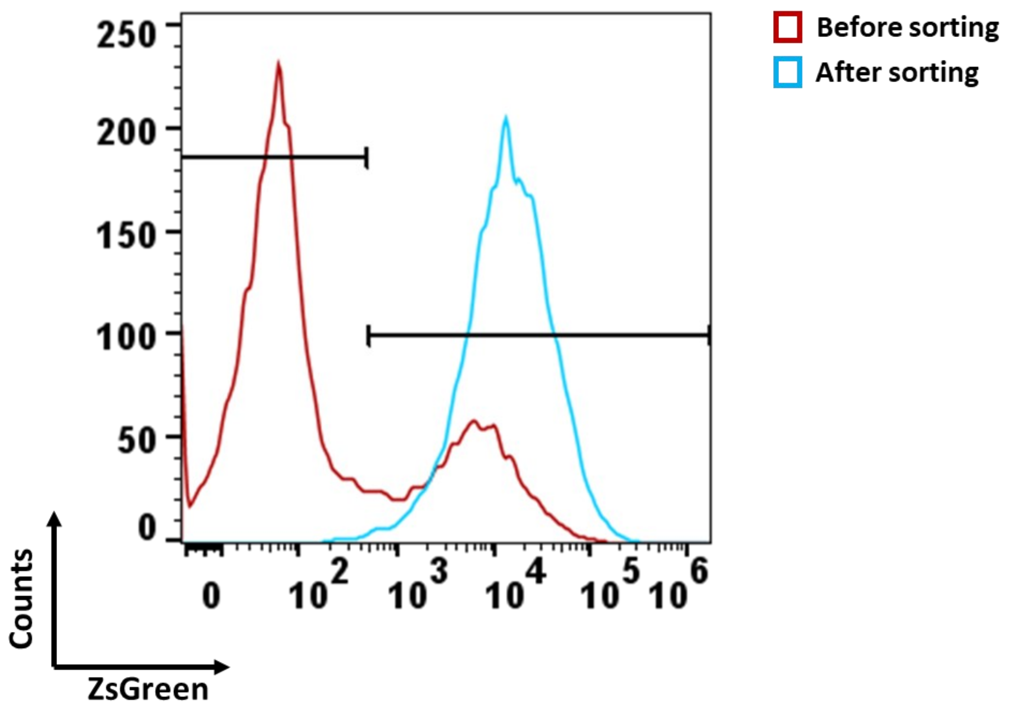

Supplemental Fig. S3. Overlay of the histograms (representative plots) showing the percentage of ZsGreen^{+} cells transduced with pZIP-MND-ZsGreen-UltramiR before and after flow based sorting.

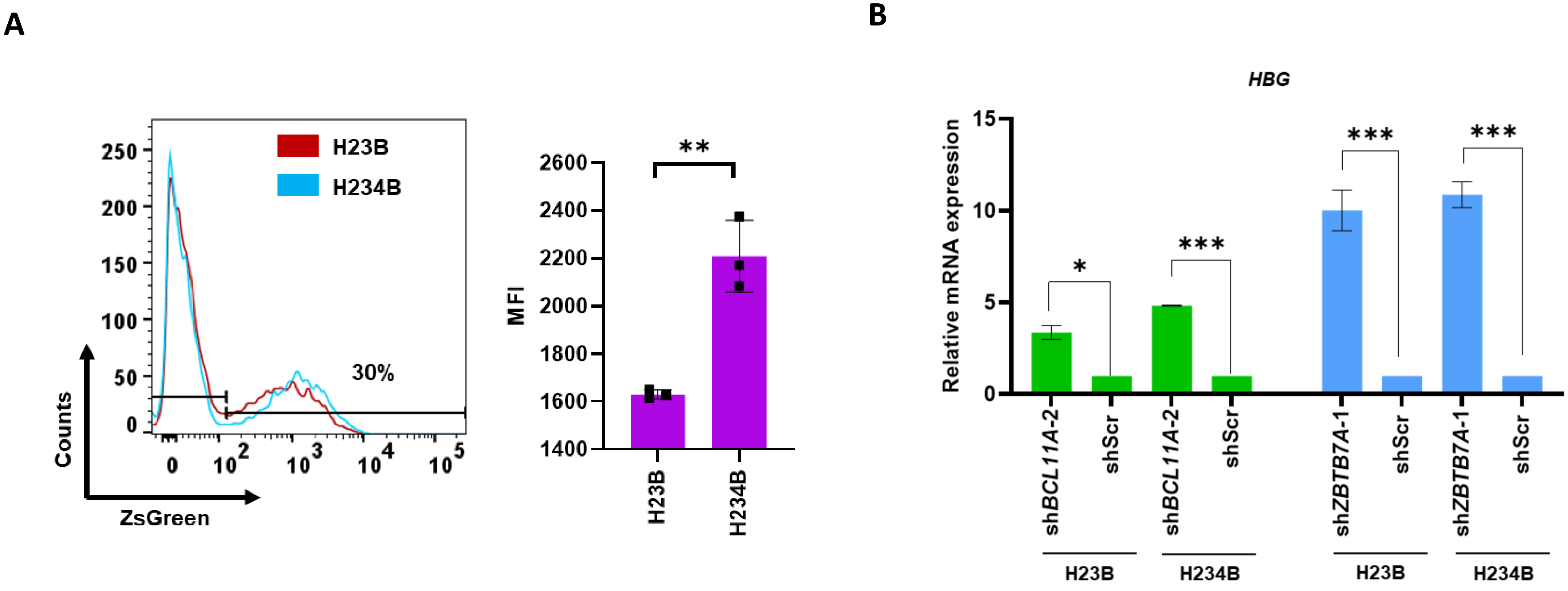

Supplemental Fig. S4. (A) Mean fluorescence intensity (MFI) of ZsGreen expression in HUDEP-2 cells transduced with H23B and H234B Ery-Lin-shRNA LVs. (B) Relative *HBG* mRNA expression after knockdown of *BCL11A* and *ZBTB7A* in HUDEP-2 cells using H23B and H234B LVs. H23B and H234B represent H23B-Ery-Lin-shRNA and H234B-Ery-Lin-shRNA LVs, respectively. Data are mean ± SD (n=3). *$p < 0.05$, **$p < 0.01$, ***$p < 0.001$.

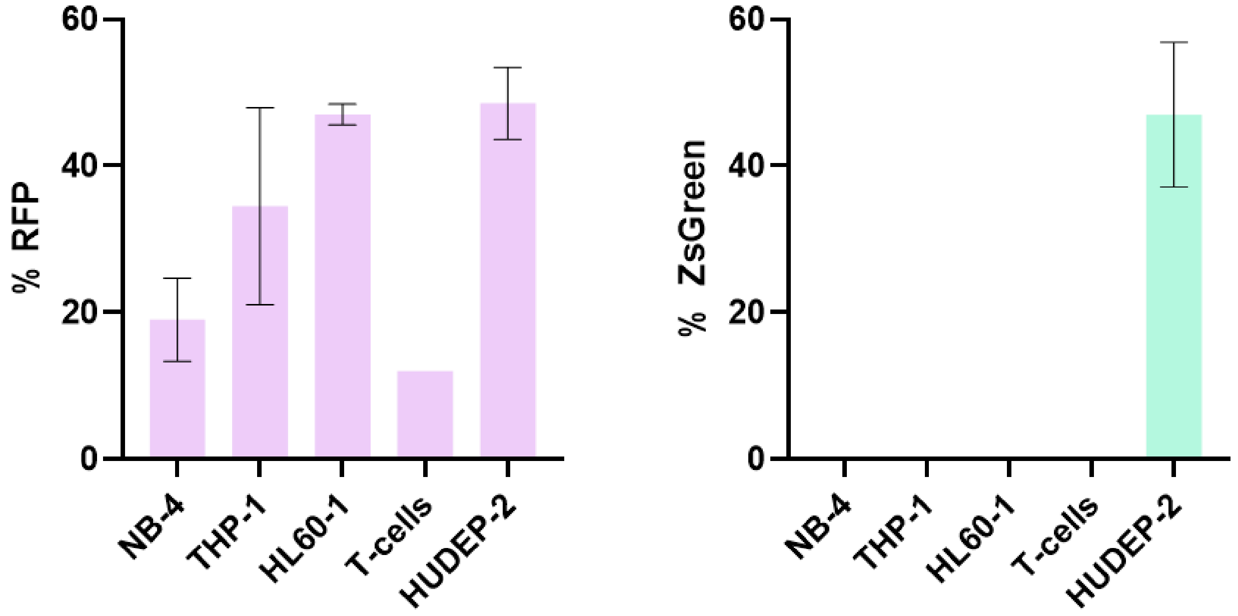

Supplemental Fig. S5. Expression of RFP (left panel) and ZsGreen (right panel) in different hematopoetic cell types after five days of transduction with H23B-Ery-Lin-shRNA and pLKO5.sgRNA.EFS.tRFP LVs . Data are mean ± SD (n=3). *$p < 0.05$, **$p < 0.01$, ***$p < 0.001$.

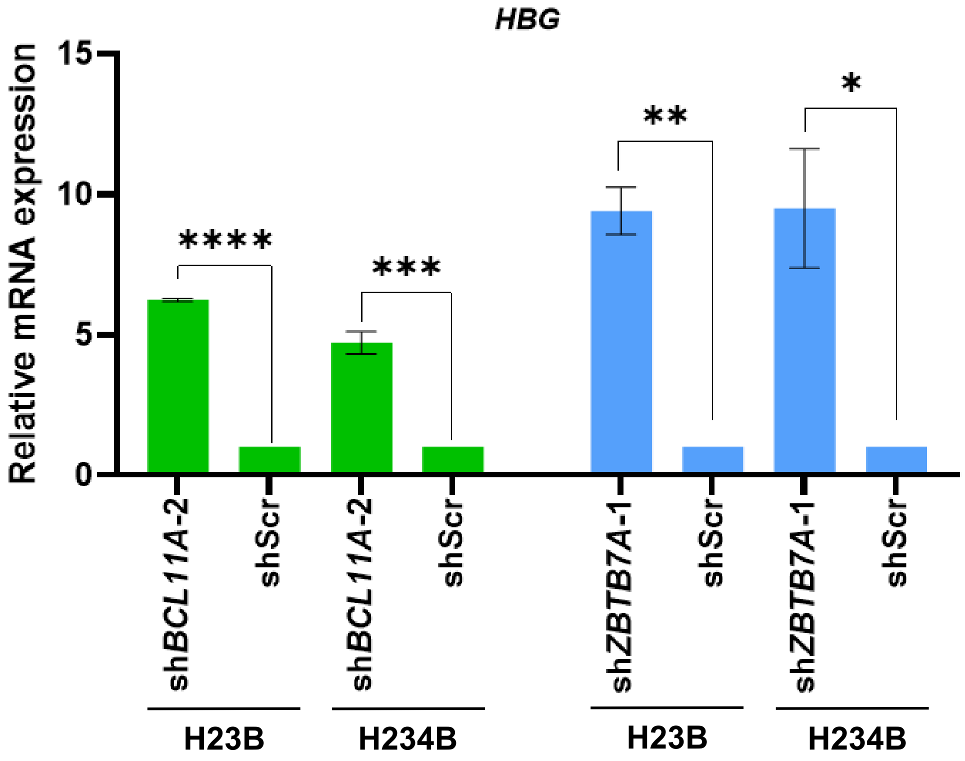

Supplemental Fig. S6. Relative *HBG* mRNA expression after knockdown of *BCL11A* and *ZBTB7A* in the flow-sorted ZsGreen$^+$ erythroid cells. Data are mean ± S.D from 4 independent experiments. *$p < 0.05$, **$p < 0.01$, ***$p < 0.001$, ****$p < 0.0001$.

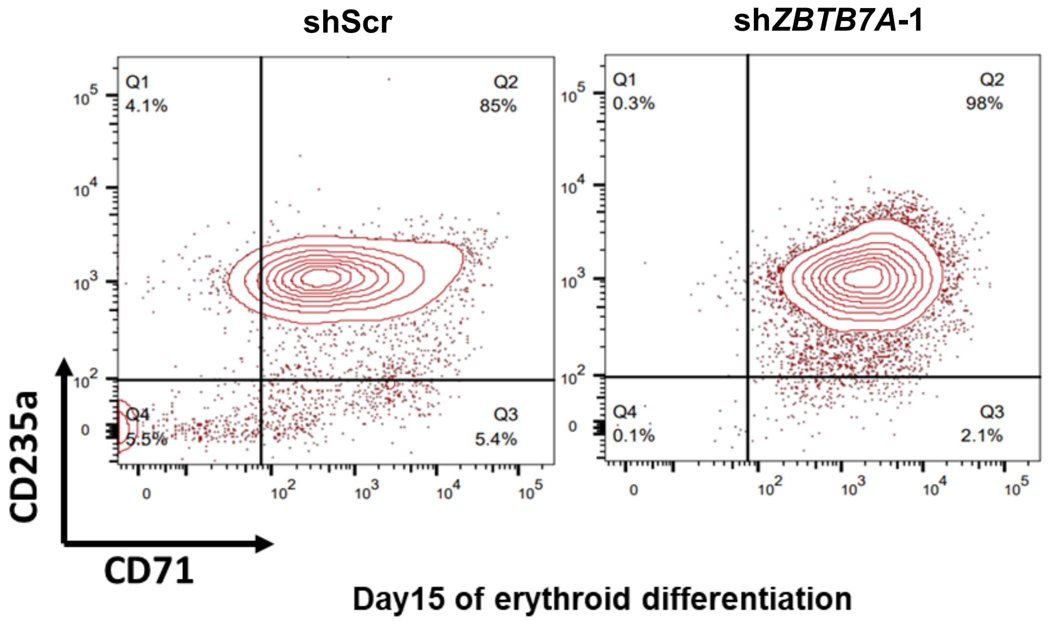

Supplemental Fig. S7. Representative flow cytometry plots of CD71$^+$CD235a$^+$ erythroid cells obtained by ex vivo erythropoiesis from CD34$^+$ HSPCs transduced with sh*ZBTB7A* and shScr.

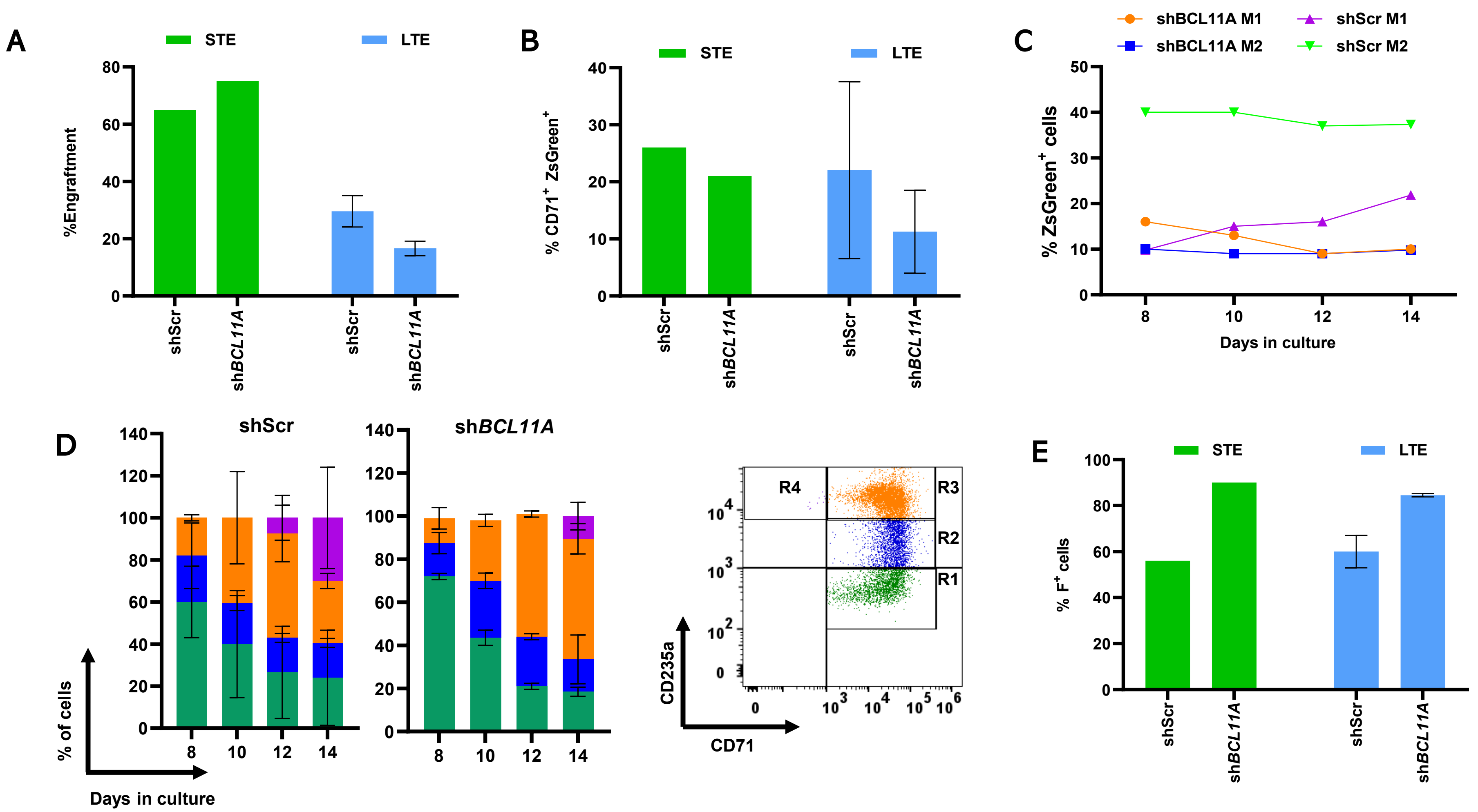

Supplemental Fig. S8. Analysis of the engrafted H23BW-Ery-Lin-shRNA transduced HSPCs in NSG mice. (A) The percentage engraftment measured in the total BM cells isolated eight weeks (short term engraftment (STE)) and 16 weeks (long term engraftment (LTE)) of transplantation. (B) The percentage of $CD71^+ZsGreen^+$ erythroid cells obtained from BM cells after 5 days of ex vivo erythropoiesis. (C) The percentages of $ZsGreen^+$ cells at various time points of erythroid differentiation shown separately for each mouse. sh*BCL11A* M1 and sh*BCL11A* M2: individual mice transplanted with H23BW-Ery-Lin-sh*BCL11A* transduced HSPCs and shScr M1 and shScr M2: mice transplanted with H23BW-Ery-Lin-shScr transduced HSPCs. (D) Erythroid differentiation kinetics in the $ZsGreen^+$ cultured erythroid cells from LTE-BM cells. R1, R2, R3, and R4 populations denote the $CD71^+$ and $CD235a^+$ expression of the transduced cultured erythroid cells on different days of ex vivo erythropoiesis. (E) The percentage of HbF^+ cells in the terminally differentiated $ZsGreen^+$ STE and LTE cells. Data are mean ± S.D from two independent experiments from LTE cells.

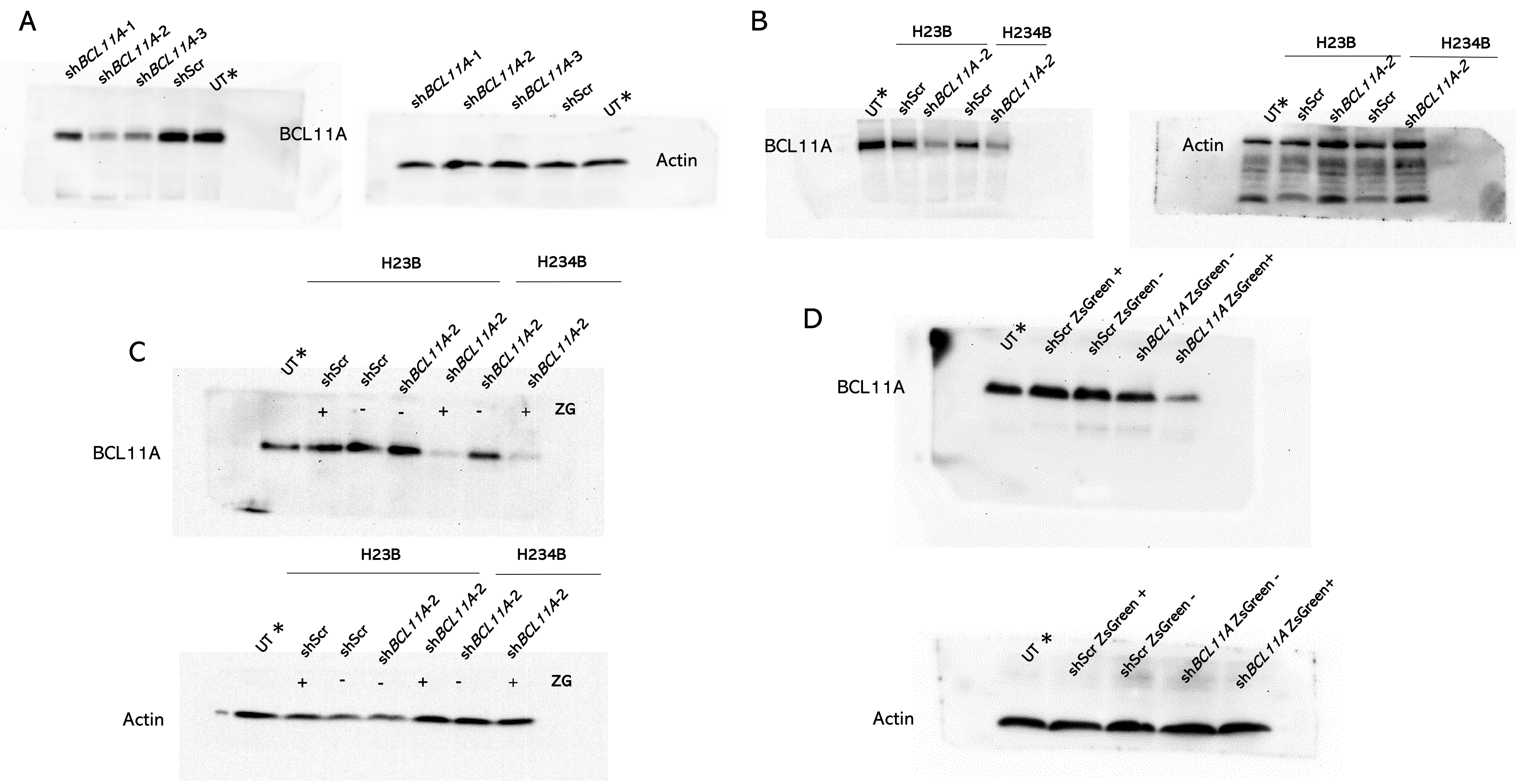

Supplemental Fig. S9. Full-length immunoblots (A) for Figure 1C, (B) for Figure 2C, (C) for figure 3B and (D) for Figure 4E. UT: untransduced. ' * ' represents lanes not shown in the main figure.

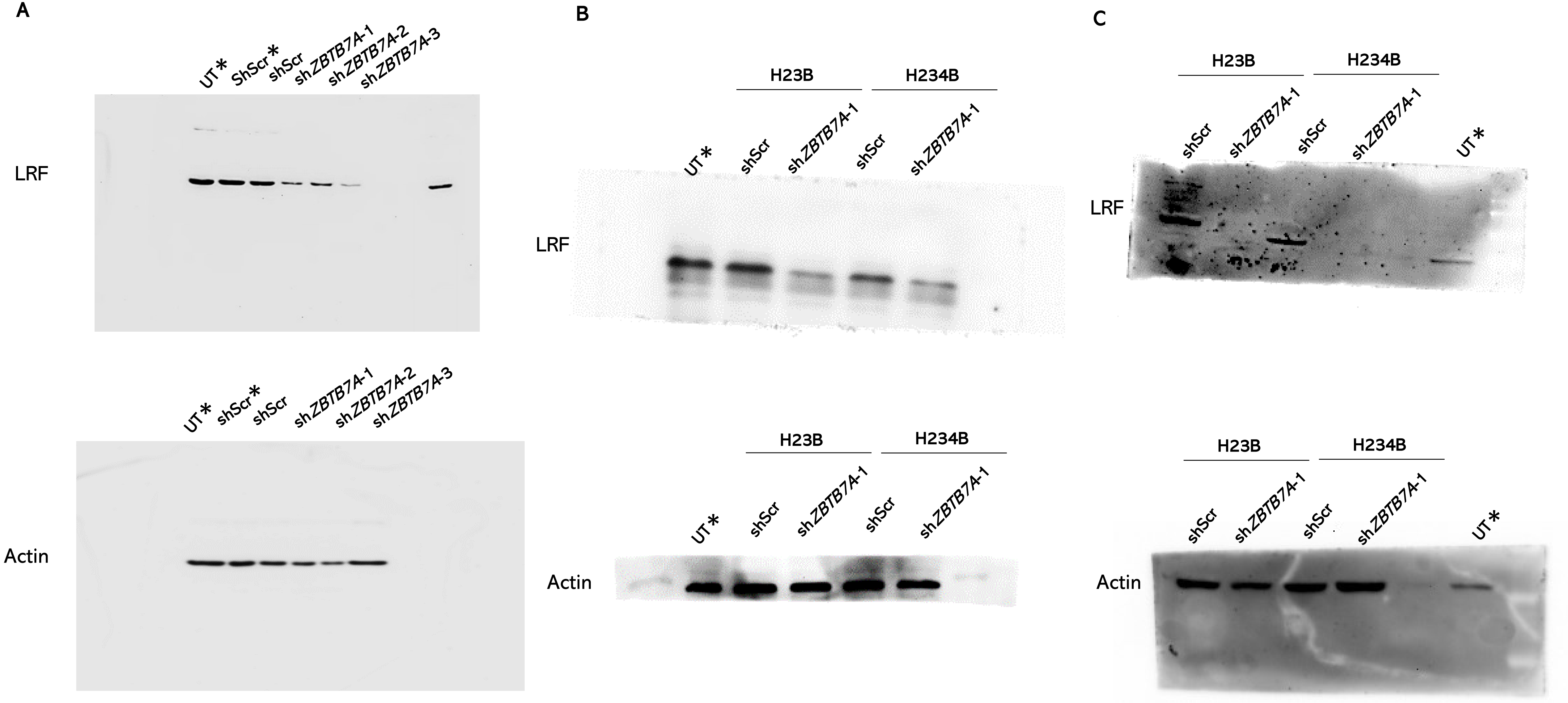

Supplemental Fig. S10. Full-length immunoblots (A) for Figure 1C, (B) for Figure 2C and (C) for Figure 3B. UT: untransduced. ' * ' represents lanes not shown in the main figure..

Supplemental Tables

Supplemental Table S1. List of oligos used in this study.

Target	Oligo Name	Sequence (5'—>3')
**Real-time qPCR**		
BCL11A	*BCL11A* RT-Forward primer	ATGCGAGCTGTGCAACTATG
	BCL11A RT-Reverse primer	GTAAACGTCCTTCCCCACCT
ZBTB7A	*ZBTB7A* RT-Forward primer	AACGTGGGTGACATCCTCAG
	ZBTB7A RT-Reverse primer	CCGTAGTGGCCGTTCTGC
**Cloning**		
mirE	mirE- Forward primer	TGAACTCGAGAAGGTATATTGCTGTTGACAGTGAGCG
	mirE -Reverse primer	TCTCGAATTCTAGCCCCTTGAAGTCCGAGGCAGTAGGC
MND	MND-Forward primer	ATCGATTAGTCCAATTTGTTAAAGAC
	MND-Reverse Primer	ACCGGTCAGATCGCGCCGAGTGAGGG
**ddPCR**		
HIV	*HIV* -Forward primer	TACTGACGCTCTCGCACC
	HIV -Reverse primer	TCTCGACGCAGGACTCG
	HIV Probe	6FAM-ATCTCTCTCCTTCTAGCCTC-BHQ-1
hTERT	*hTERT* -Forward primer	GGCACACGTGGCTTTTCG
	hTERT -Reverse primer	GGTGAACCTCGTAAGTTTATGCAA
	hTERT Probe	HEX-TCAGGACGTCGAGTGGACACGGTG-BHQ-1

Supplemental Table S2. List of antibodies used for flow cytometry analysis.

Antibody	Flurochrome	Vendor	Catalogue No.
hCD45	FITC	BD Pharmingen	555482
mCD45.1	PERCP-CY5.5	BD Pharmingen	560580
hCD3	APC	BD Pharmingen	340440
hCD19	PE	BD Pharmingen	340364
hCD13	APC	BD Pharmingen	555394
hCD33	PE	BD Pharmingen	340474
hCD71	APC	BD Pharmingen	551374
CD235a	PE	BD Pharmingen	555570
HbF	APC	ThermoFisher Scientific	MHFH05

Supplemental Reference:

1. Brendel, C. et al. Lineage-specific BCL11A knockdown circumvents toxicities and reverses sickle phenotype. J. Clin. Invest. 126, 3868–3878 (2016).
